# Printing Hybrid, Interdigitated Back-Contact Solar Cells

**DOI:** 10.3390/ma19050985

**Published:** 2026-03-04

**Authors:** Guancheng Li, David Angel Trujillo, Robert L. Opila

**Affiliations:** Materials Science and Engineering, University of Delaware, Newark, DE 19716, USA; guanchl@udel.edu (G.L.);

**Keywords:** solar cells, photovoltaics, interdigitated back-contacts, PEDOT:PSS, heterojunction, inkjet printing

## Abstract

Interdigitated back-contact solar cells were fabricated entirely with inkjet printing. poly(3,4-ethylenedioxythiophene):polystyrene sulfonate (PEDOT:PSS), TiO_2_, and metal lines were printed on a textured silicon substrate with only one inkjet printer. No vacuum deposition or diffusion of a back surface field is needed for the printed IBC solar cell. Adding co-solvent to the PEDOT:PSS and passivation of the Si surface significantly reduced the losses and enhanced the short-circuit current, Jsc, and, as a result, improved the fill factor and efficiency of the devices. The thickness of the PEDOT:PSS layer is approximately half a micrometer measured by profilometer, which is thicker than the optimal range typically reported; there is still a best short-circuit current, Jsc, of 19.2 mA/cm^2^. To further improve the performance of the devices, an anti-reflective coating on the front side is required. Also, an improved metal contact ink is needed to improve the contact resistance between the PEDOT:PSS layer and the metal contact. The initial performance of all printed cells are compared to conventionally fabricated devices.

## 1. Introduction

The growing global energy needs and environmental concerns have encouraged the search for efficient, cost-effective, and sustainable photovoltaic (PV) technologies. Interdigitated Back-Contact (IBC) solar cells offer significant performance advantages over traditional architectures due to the reduced shadowing loss [1]. However, their fabrication typically relies on complex, multi-step lithographic processes [1,2,3]. A compelling pathway to simplify this manufacturing is through solution-based techniques. Currently, IBC solar cells hold a smaller market share compared to mainstream conventional solar cells due to their complex manufacturing and concomitant increased cost. Nevertheless, their market share is growing rapidly, particularly in residential and other value-driven applications, such as concentrator photovoltaics (CPV) and solar race cars. IBC solar cells are predicted to have at least 13% revenue share worldwide in 2028 based on the International Technology Roadmap study [4].

Among different types of solar cells, we are particularly interested in hybrid solar cells because they represent a synergy of high-performance inorganic materials and low-cost organic materials [5]. By combining the well-established, excellent electronic properties of crystalline silicon with the versatile, solution-processable nature of organic materials [6,7,8], we can create devices that are both highly efficient and potentially simpler and cheaper to manufacture, which are perfectly aligned with the performance potential and manufacturing challenges of IBC solar cells.

Solution-based processing methods, like spin-coating, have been used to deposit organic layers for hybrid solar cells, such as poly(3,4-ethylenedioxythiophene):polystyrene sulfonate (PEDOT:PSS) with co-solvent ethylene glycol (EG) as the hole transport layer (HTL) on Si wafers [9]. However, these techniques, not only spin-coating, but doctor blading [10], screen printing [11], and flexographic printing [12], inherently lack the patterning capability required for IBC’s fine interdigitated features. Consequently, additional patterning steps to achieve the needed structure are often required for those IBC solar cells manufactured with solution-based methods. For example, Lv et al. used spin-coated Nafion film as a passivation and anti-reflection layer, PEDOT:PSS film as HTL, but also used additional plasma etching to fabricate a patterned lithium acetate film as an electron transport layer (ETL), and used thermal evaporation to deposit the metal contacts; the resultant IBC solar cell showed a power conversion efficiency (PCE) of 15.4% [13]. In another example, Lin et al. used spin-coated PEDOT:PSS film as HTL, but E-beam evaporation, atomic layer etching (ALD), and plasma-enhanced chemical vapor deposition (PECVD) were also required to deposit MgOx as the ETL, and Al_2_O_3_/S_i_N_x_ bilayer film as a passivation and antireflection layer for the front side, respectively; the obtained IBC solar cell showed a PCE of 16.3% [14]. In reports that use solution processing to fabricate IBC, solar cells require additional physical processing techniques to complete the solar cells. In this work, we will show that it is possible to fabricate a solar cell using entirely solution processing.

Inkjet printing is emerging as a promising alternative that addresses the specific needs of IBC solar cells by combining solution processing with direct digital patterning and allowing 2D freedom-of-design. Previous research has applied inkjet printing to traditional solar cell structures, such as organic solar cells (OSCs) and perovskite solar cells (PeSCs); see examples in reference [15]. Groen et al. achieved fully inkjet-printed OSCs with the structure of Ag/PEDOT:PSS/ZnO/P3HT:PC60BM/PEDOT:PSS/Ag and achieved a PCE of 1.7% [16]. Pesch et al. were able to obtain uniform perovskite films by introducing a dimethyl sulfoxide (DMSO) vapor treatment step on the evaporated lead iodide film, which ultimately achieved a PCE of 18.2% [17]. But there are only a few studies focused on fabricating IBC solar cells with inkjet printing. Often inkjet printing is used as a complementary tool to help deposit a small fraction of the device. For example, Takagishi et al. used an inkjet-printed etch mask to form IBC patterns [18]. Wehmeier et al. prepared the in situ doped and structured passivating contacts with inkjet-printed silicon ink [19]. And Hsiao et al. used inkjet-printed and patterned PEDOT:PSS as the seed layer for the subsequent Cu electroplating [20].

In this work, we aim to fully exploit the simplicity of this method by demonstrating a fully inkjet-printed hybrid IBC solar cell, depositing all key layers using a single printer to streamline the fabrication process. This technique is not only simpler but also ideal for developing conformable solar cells [15]. Although the inkjet printing technology and the printed devices are currently limited by the printing resolution and carrier recombination loss at the front surface, we will show in this work that this method holds significant potential for scalable and versatile solar cell manufacturing once these challenges are addressed. The performance of printed cells will be compared to conventionally fabricated devices as noted.

**Figure 1:**
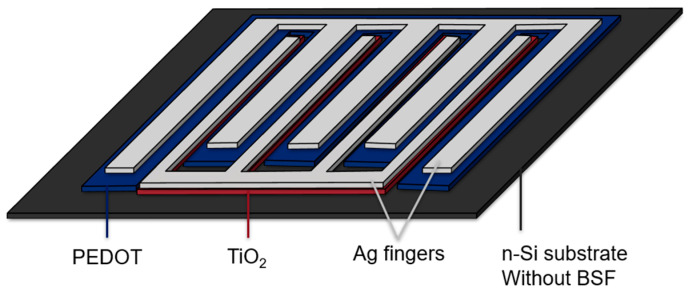
Schematic of the interdigitated back-contact cell. The blue region represents the PEDOT:PSS. The red region shows the TiO_2_. The gray region shows the electrically isolated Ag fingers. All these materials are on the contact side (rear). Light is absorbed through the side of the device that has no metallization; that is the bottom (front/illumination side) of the Si cell shown above. The illuminated side is referred to as the front of the device.

In Figure 1, a schematic of our printed IBC cell is shown. The blue region represents the PEDOT:PSS which acts as a hole selective contact. The red region shows where the TiO_2_, an electron selective material on silicon, is deposited. Electrically isolated Ag-ink fingers are deposited on both the TiO_2_ and the PEDOT:PSS. Light is absorbed through the bottom of the Si cell. In the printed cell there are six PEDOT:PSS fingers, each approximately 1.3 mm wide, and five TiO_2_ fingers, each about 0.3 mm wide.

In this work, a state-of-the-art nano-dispensing printer was used [21]. This permitted a hybrid solar cell with PEDOT:PSS as HTL to be printed. Currently this can be done with spin coater to deposit the PEDOT:PSS layer and electron beam evaporator to deposit the metal contacts on both sides, respectively. However, now one inkjet printer can fabricate both the PEDOT:PSS layer and metal contacts. In addition, with inkjet printing, different patterns of PEDOT:PSS can be achieved, making it a promising method for fabricating all the functional layers of IBC solar cells. This cannot be done by spin coating. Also, compared with the inkjet printer, the electron beam evaporator requires substantially more energy and a better controlled environment such as a cleanroom. This paper will address the technical challenges of employing an inkjet printing method for deposition of the PEDOT:PSS layer, TiO_2_ layer, and metal contacts and illustrate that it is a promising method for fabricating IBC solar cells.

## 2. Materials and Methods

N-type textured wafers were provided by Solar Power Labs at the Arizona State University. Random pyramidal texturization was performed on both sides using alkaline potassium hydroxide (2% KOH yielding pyramid sizes of about a 3–5 µm base size). Texturing minimizes energy loss by reflection [22,23]. The wafers were 145 microns thick with a resistivity of 1–5 Ω·cm.

The PEDOT:PSS was purchased from Heraeus (Vandalia, OH, USA) and the Clevios PH1000 blend (Heraeus, Vandalia, OH, USA) was used as is. Ethylene glycol (EG) and dimethyl sulfoxide (DMSO) were purchased from Sigma Aldrich (St. Louis, MO, USA) and used as co-solvents. Capstone FS-3100 (Dupont, Wilmington, DE, USA) was used as the surfactant in the ink. Benzoquinone (BQ) purchased from Sigma Aldrich was dissolved in methanol (ME) to prepare 0.01 M BQ/ME solvent as passivation for the hydrogen terminated silicon surface [24].

Wafers were cleaned following procedures described by Opila and Teplyakov et al. in the fume hood [25]. First, the wafers were merged into the Piranha solvent (H_2_SO_4_:H_2_O_2_ = 4:1) for 5 min, followed by a five-minute deionized water (DIW) rinse and a two-minute immersion in hydrofluoric acid (HF, 2 vol%). After HF dipping, wafers were rinsed in DI water for 2 min to remove any trace HF and then blow-dried with nitrogen. After cleaning, the wafers were immersed in BQ/ME solution for an hour then dried and immediately transferred to a vacuum container before inkjet printing.

In this work, an N-Scrypt 3Dn-300 printer (N-Scrypt, Orlando, FL, USA) was used. It is a hybrid additive/subtractive system that employs multiple tools to realize complex structures in a single workflow. These include (but are not limited to) the SmartPump (nano-dispensing printer), the nMill (high-speed micro-milling), the nFD (fused deposition modelling) and the Pick-n-Place (component placement) tools [26]. The precision manufacturing capability of the SmartPump tool has opened new design spaces for the fabrication of solar cells [21].

The Nscrypt 3Dn-300 system was used as the inkjet printer for this project. Nozzle with diameters of 75 µm and 100 µm were used for printing PEDOT ink (PEDOT:PSS mixed with 7 vol% co-solvent and 0.5 vol% surfactant) and silver ink (Dupont), respectively.

All the printings are done under room temperature and ambient humidity. Detailed printing parameters will be discussed in the following section. After printing each layer, an annealing step in the air is applied under different conditions. PEDOT:PSS and TiO_2_ layers were annealed under 120 °C for 15 min and Ag contacts were annealed under 100 °C for an hour.

The devices were tested using illuminated current density–voltage measurements (JV). JV response was measured using a DC source meter (2400 Sourcemeter, Keithley, Cleveland, OH, USA) in the dark and light in both scan directions with a scan rate of 0.1 V/s under air mass 1.5 G standard illumination (calibrated by the current output of reference silicon solar cell). No hysteresis was observed in any of the devices at this scan rate. A 10 mm × 10 mm mask was applied to define the active area. The Keithley multimeter is a precision Source Measure Unit (SMU) that combines a voltage source, current source, voltmeter, ammeter, and ohmmeter in a single instrument, widely used for semiconductor testing, materials research, and device characterization.

For comparison front-contact devices were fabricated. For these devices, an additional back surface field (BSF) was fabricated on the rear end of part of these wafers via diffusion. Phosphorous oxychloride diffusion was performed at 820 °C for 15 min with a POCl_3_ carrier gas at a flow rate of 1500 sccm, which led to phosphosilicate glass (PSG) growth and dopant drive-in. Finally, buffered oxide etching was performed for 10 min to remove the PSG. The sheet resistance of the BSF side is close to 55 Ω/square.

## 3. Results and Discussion

### 3.1. Inkjet Printing of PEDOT:PSS Layer

The thickness of the PEDOT:PSS layer plays a crucial role in the performance of hybrid heterojunction solar cells. For conventional front-contact hybrid solar cells, the thickness of the PEDOT:PSS layer affects the anti-reflective properties of the solar cell. Optimal thickness is necessary to minimize reflection and maximize light absorption. Previous simulations performed using the online optical simulator OPAL 2 studies suggested an optimal thickness of around 65 nm on a planar silicon substrate for the best anti-reflective properties under AM1.5G illumination conditions [27]. Meanwhile, the transverse conductivity of the PEDOT:PSS layer increases as the thickness increases [28]. For back-contact solar cells, the PEDOT:PSS layer thickness has minimum effect on light adsorption [29]. However, because of its limited vertical hole transport efficiency (conductivity across the PEDOT:PSS film), the favorable film thickness would still be in the same range as the front-contact solar cells, tens of nanometers [30]. Moreover, based on the electrical insulating properties of PSS, the addition of co-solvents like ethylene glycol (EG) and dimethyl sulfoxide (DMSO) are still needed to improve the conductivity [31], but the thickness must still be carefully controlled to balance conductivity along the film and across it.

Thicker PEDOT:PSS layers may be sensitive to humid environments [32]. The hygroscopic nature of PEDOT:PSS can lead to increased moisture absorption, which can effectively de-dope the polymer and increase the series resistance of the solar cell, leading to the formation of s-shaped JV curves with much reduced fill factor [32].

When using spin-coating techniques to deposit PEDOT:PSS layer, the thickness can be adjusted by changing the spinning speed and the amount of ink use. However, it is a little more complicated in inkjet printing techniques. The thickness of the PEDOT layer in inkjet-printed solar cells is influenced by four parameters: printing speed, nozzle size, pumping pressure and ink properties Their optimization is essential for achieving a thin, uniform layer and avoids delamination during annealing.

Printing speed directly affects the amount of ink deposited per unit area. Higher printing speeds reduce the residence time of the ink on the substrate, leading to thinner layers. However, excessively high speeds can cause incomplete droplet coalescence, resulting in pinholes and reduced layer uniformity. Here we chose the 5 mm/s as the printing speed.

In addition to printing speed, nozzle diameter is another critical factor. Smaller nozzles produce finer droplets, which naturally result in thinner layers. However, the main composite in PEDOT ink was high viscosity water-based PEDOT:PSS solution and water evaporation also occurs during printing, which made it more prone to clogging. As a result, a 75 µm-size nozzle was chosen and the PEDOT solution was filtered using a 10 µm filter before use.

Pumping pressure influences the velocity and volume of ink droplets ejected from the nozzle. Lower pumping pressures reduce droplet velocity, minimizing splashing and ensuring a more controlled deposition that would not spread too much and ruin the interdigitated pattern. This results in thinner and more uniform layers. However, pressures that are too low (<0.5 psi) can lead to incomplete printing and any slight amount of evaporation could lead to clogging, particularly in complex patterns like interdigitated contacts. As a result, a pressure of around 1.0 psi is usually used in these experiments, and slightly adjusted based on different compositions of ink. For example, 0.8 psi was used without a co-solvent.

Finally, different ink compositions have different viscosity and wettability, which leads to different printing parameters and layer thickness. For example, the addition of surfactant can significantly enhance the ink wettability on H-terminated silicon wafer, which can increase the printing width and decrease the ink amount use.

When these factors are optimized, their combined effect significantly enhances the ability to produce thin PEDOT:PSS layers. There is also an adhesion issue at the interface between the hydrophobic H-terminated silicon wafer and the water-based PEDOT ink. If the PEDOT:PSS layer is too thick, it may detach from the silicon wafer during the annealing process. With the optimized printing parameters, the adhesion issue was successfully solved. However, due to the inherent properties of the ink, the adjustment of these parameters is constrained, resulting in a thickness of approximately 0.5 μm as measured with a profilometer, making it difficult to achieve a nanoscale thickness. The variation in thickness at the center of the deposited lines was approximately 0.1 micrometer.

**Figure 2:**
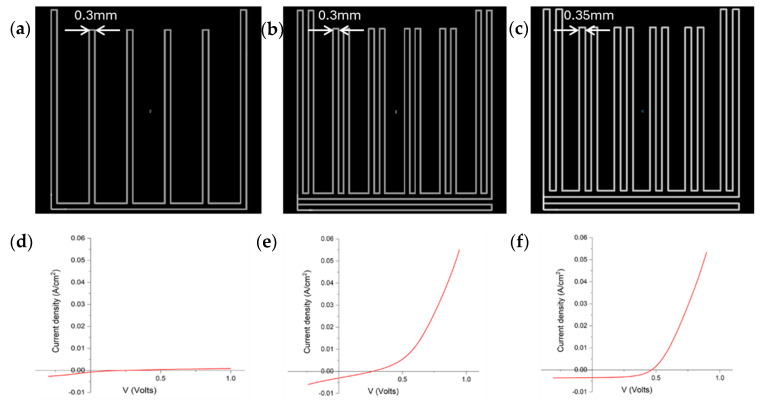
(**a**–**c**) Printing pattern for PEDOT:PSS layer with pitch size (distance between two adjacent fingers) of 2 mm. The spacing between the lines is noted in the diagram. Patterns individually printed in each finger flow together for an overall width of approximately 1.3 mm. (**d**–**f**) JV performance of devices with PEDOT:PSS printed with patterns (**a**–**c**), respectively.

Another way to improve the devices’ performance is to increase the coverage of the PEDOT:PSS on the silicon substrate. This value is determined by the percentage of the rear surface that is covered by PEDOT:PSS. PEDOT:PSS is known as a conductive polymer that can effectively collect and transport photogenerated carriers [33]. Increasing its coverage area can expand the range of carrier collection, reduce recombination losses, and thereby improve cell efficiency. Also, since PEDOT:PSS forms good ohmic contact with the silicon wafer, increasing its coverage area optimizes contact and reduces interface resistance. Although increasing PEDOT:PSS coverage does not significantly affect light adsorption, it allows for a more uniform collection of photogenerated carriers, thereby improving light energy utilization. Three of the patterns used for printing of the PEDOT:PSS layer are shown in Figure 2. The lines drawn following the closely spaced patterns flow together, forming one finger. For Figure 2a,b, the interval measured between adjacent lines was found to be 0.3 mm, while it was 0.35 mm in Figure 2c. We calculated the coverage by measuring the average width of a couple of printed lines by profilometer and then calculating the area of the pattern to get the coverage.

If we set the illumination area as 10 mm × 10 mm, the coverage of the PEDOT:PSS was measured to increase from less than 40% in Figure 2a to around 80% in Figure 2c. The influence of PEDOT:PSS coverage shown in this work agrees with the simulated results done by ATLAS in previous work [34]. Increases in short-circuit current, Jsc, and efficiency are obtained with increasing coverage of PEDOT:PSS. This effect is mainly due to the reduction of recombination loss at rear side region without emitter coverage by increasing the PEDOT coverage ratio. This also explains why changing the emitter coverage ratio has a stronger effect on low-level passivation devices [34,35]. These results are summarized in Table 1. Because of the performance of the pattern in Figure 2c, it was used in all studies described below. Any short circuits between the electron contacts and the hole contacts would show up in the J-V curves as straight Ohmic lines.

### 3.2. Effect of Ink Composition

The addition of co-solvents to the PEDOT:PSS solution significantly enhances the performance of solar cells by overcoming the key challenges of PEDOT:PSS solvent related to electrical conductivity, film formation, optical properties, and interfacial quality [9]. PEDOT:PSS is a composite of conductive PEDOT and insulating PSS, which means a reduced conductivity due to the presence of PSS [36]. Co-solvents such as EG and DMSO can modify the microstructure of PEDOT:PSS, promote better interaction between PEDOT chains and thereby increase the conductivity. This improvement in conductivity facilitates efficient hole transport, reducing series resistance of the solar cell [37]. Additionally, surfactants improve the wettability and flowability of the PEDOT:PSS solution, enabling the formation of uniform and conformal films, particularly on textured silicon substrates. This uniformity minimizes defects such as pinholes, which can act as recombination centers, and ensures consistent performance across the device. By minimizing defect states at the interface, co-solvents also help lower recombination losses, resulting in higher open-circuit voltage (Voc) [9]. For stability, PEDOT:PSS is inherently hygroscopic, absorbing moisture from the environment, which can degrade its performance over time [38]. Co-solvents improve the moisture resistance of PEDOT:PSS, enhancing the long-term stability of the solar cell. They also modify the microstructure of PEDOT:PSS, making it more stable under high-temperature or humid conditions, which is critical for real-world applications [39,40]. Overall, the use of co-solvents in PEDOT:PSS solutions is essential for optimizing the electrical, optical, and interfacial properties of PEDOT-related solar cells, leading to higher efficiency, stability, and reliability.

Although adding co-solvent can greatly improve the performance of solar cells, it can cause problems during printing IBC solar cells. If only EG or DMSO are added as co-solvents for higher conductivity, a loss of adhesion between the silicon wafer and the PEDOT:PSS layer often occurs. The addition of EG or DMSO can also induce phase separation between PEDOT and PSS, altering the microstructure of the film, as we mentioned above. This phase separation may create inhomogeneous regions within the PEDOT:PSS film, making it more prone to peeling off from the silicon substrate [41]. Also, during the annealing process, the co-solvent evaporates rapidly, which can cause the PEDOT:PSS film to shrink. This shrinkage generates internal stress at the interface, weakening the adhesion between the film and the substrate. Since the thickness of PEDOT:PSS layer is much higher than the spin-coated devices, the detachment problem occurs more easily. Adding surfactant as a co-solvent can improve the wettability of the PEDOT:PSS ink on the silicon wafer, which can solve the detachment problem in general. In our previous work, Triton X-100 was used as the surfactant for the spin-coated hybrid solar cells because of its enhancement of wettability on a hydrophobic surface, but it is too strong and makes the ink easily spread when printing specific patterns such as these interdigitated ones. As a result, Capstone FS-3100, a nonionic fluorosurfactant, was used to limit excessively strong increasing wettability.

With these efforts, we successfully achieved an interdigitated patterned PEDOT:PSS layer with co-solvent and surfactant by inkjet printing processing. Comparing the results in Table 1, the Jsc largely increased substantially by adding DMSO and surfactant to reduce the negative effect of PSS and enhance the interface quality. Overall, these changes lead to better power efficiency.

### 3.3. Electron Transport Layer Printing

The Electron Transport Layer (ETL) is a fundamental component in solar cells, particularly in IBC solar cells, where its role is critical for achieving high efficiency and performance. The primary function of the ETL is to facilitate the efficient extraction and transport of electrons generated in the active layer while blocking holes to minimize recombination losses. In IBC solar cells, this task becomes even more challenging due to the unique architecture where both electron and hole contacts are located on the back side of the cell. The ETL must ensure that electrons are effectively collected at the back contact without interference from the hole transport layer (HTL), requiring precise material selection and fabrication techniques to maintain optimal energy-level alignment and minimize interface defects.

One of the key challenges in fabricating ETLs for IBC solar cells is material selection. Commonly used materials include metal oxides like TiO_2_, ZnO, and SnO_2_, organic materials such as PCBM and C_60_, and perovskites like CsPbBr_3_ [42,43,44,45,46,47,48]. Each material has its advantages, such as high electron mobility or solution processability, but also faces limitations, such as long-term stability or the need for demanding conditioning treatment, such as high-temperature processing. For example, SnO_2_ is a promising ETL material because of its high electron mobility, wide band gap, and long stability under UV illumination. However, to achieve a better bandgap matching to facilitate the charge extraction and reduce interfacial recombination, an extra surface treatment such as TiCl_4_ treatment is required [49,50,51]. ZnO, a transparent conductive oxide, is a prominent ETL material. However, its sensitivity to humidity and acidic environments would be an issue [48]. Also, the post-treatment with acidic solutions for some of the precursors may destroy the other layers of the solar cells. Achieving the right balance between performance, stability, and process compatibility is important [52].

The fabrication process itself presents further challenges, particularly in achieving uniform deposition and precise patterning on the back side of IBC solar cells. Techniques such as spin-coating, ALD, sputtering, and chemical bath deposition (CBD) are commonly employed, each with its own set of limitations. For instance, spin-coating is simple but not suitable for IBC solar cells, while ALD offers precise control but is costly and slow. Inkjet printing, despite the difficulty of selecting suitable materials that are printable on the silicon wafer, is low-cost and promising for patterning. Moreover, the processing temperature also needs to be considered, as many high-performance ETL materials require annealing at elevated temperatures that could degrade the PEDOT.

Here we chose TiO_2_ as the ETL material because of its high electron mobility and good energy-level alignment with silicon [53,54]. To get the TiO_2_ nanoparticle ink, 75 μL of TiO_2_ nanoparticle suspension (Sigma-Aldrich, 40 wt% in water, ~21 nm) was mixed with 1885 μL of DI water and 40 μL of titanium diisopropoxidebis(acetylacetonate) (Ti(acac)_2_OiPr_2_, Sigma-Aldrich, 75 wt%). Then the suspension was stirred overnight before use [52]. During printing, the TiO_2_ ink was printed using a similar setup to the PEDOT ink, but with a lower printing pressure at 0.5 psi. The printing path was programmed to fit in the gaps between the existing PEDOT:PSS fingers precisely as the red area shown in Figure 1. With the printable TiO_2_ ink, we successfully achieved an inkjet-printed TiO_2_ layer with the pattern for IBC solar cells. Here we compared the solar cells with and without an ETL on both front-contact and back-contact devices (shown in Table 1).

As shown in the table, by applying the printable TiO_2_ ETL ink with proper band alignment between the conduction band of ETL and silicon wafer, the Voc of the devices significantly increased. According to the correlation between Voc and current given by Equation (1), the increase of Voc results from the reduction of the reverse saturation current (J0). This indicates that the addition of proper ETL facilitates efficient electron extraction while blocking holes, thereby reducing the carrier recombination loss at the surface and, as a result, enhancing the efficiencies of the devices.

(1) $$V_{OC}=\frac{kT}{q}ln\left(\frac{J_{SC}}{J_{0}}+1\right)$$

where q is electron charge, k is Boltzmann’s constant, and T is temperature.

### 3.4. Surface Passivation and Recombination

Our group has previously shown that benzoquinone (BQ) is an effective surface passivation for silicon wafers. It can modify the silicon surface and reduce surface recombination, which is critical for enhancing the performance of solar cells [55]. The principle of benzoquinone passivation is through its chemical interaction with the silicon surface. It reacts with dangling bonds and defects of H-Si to form a stable passivating layer. When used in a solution with methanol (BQ/ME), benzoquinone interacts with the hydrogen termination of the silicon surface, effectively passivating dangling bonds and significantly lowering the rate of recombination at the surface, which is evaluated by the surface recombination velocity (SRV). This reduction in SRV, as shown with implied Voc, is essential for minimizing charge carrier recombination losses, which directly impacts the efficiency of solar cells [55].

One of the advantages of benzoquinone passivation is its ability to achieve low SRV values at room temperature on bare Si wafers, as low as 1.6 cm/s, which is comparable to some high-temperature passivation methods [24]. Additionally, benzoquinone passivation is performed at room temperature and does not introduce additional complexity to the manufacturing process, making it a low-temperature and simple process that will not strongly affect the surface properties, such as the interfacial energy. This suggests that this passivation technique is compatible with inkjet printing.

In this work, benzoquinone passivation played a measurable role for the printed devices. The passivation effect enhances the minority carrier lifetime, leading to better charge collection and higher Jsc (shown in Table 1). Benzoquinone passivation also ensures a high-quality interface between the silicon substrate and the PEDOT:PSS layer. This modification of the interface reduces losses and improves the overall efficiency of the device. Also, the increased short-circuit current after the addition of the passivation process suggests that the recombination loss at the PEDOT:PSS/silicon interface could be the primary issue existing for this IBC structure solar cell.

The long term stability of benzoquinone passivation was not tested in the current work. Since the effectiveness of the passivation depends upon the oxidation of the surface, in ambient conditions, it is only effective for a few days. In production, the device would have to be hermetically sealed soon after fabrication.

### 3.5. Inkjet-Printed Metal Contacts

DuPont™ KA802 silver ink was used to print the metal contact for both electrodes. It is a polyimide based conductive silver ink designed for high-performance printed electronics, particularly in applications requiring flexibility, adhesion, and high conductivity (<20 mΩ/sq/mil). Its low curing temperature (around 90–150 °C) makes it suitable for heat-sensitive materials, like PEDOT:PSS in this work.

Although this ink can be applied in the 3D printing of solar cells, its intrinsic properties may still have certain negative impacts on the performance of solar cells. As is well known, the conductivity of inks is much worse than the pure metal. Also, it is possible that contact resistivity of the printed Ag/PEDOT:PSS interface with the silver ink used in this work is larger than the contact resistivity of the electrodes fabricated with electron beam Ag physical vapor deposition on front-contact devices in our previous work [9]. As shown in the last two entries in Table 1, Jsc was significantly decreased by only changing the evaporated Ag to the printed one, decreasing the fill factor and the power efficiency proportionately. The silver ink does contain polymer composites that need to be burned out after printing, which may make the interface quality worse.

### 3.6. Future Work

IBC solar cells in this work, fabricated with fully inkjet printing, still have relatively poor device performance, especially a low short-circuit current. Although textured silicon wafers were used to enhance the anti-reflective properties of this device, their performance is still worse than the front-contact devices fabricated in our previous work, where a PEDOT:PSS layer also plays a crucial role as anti-reflective coating [56]. As a result, a proper anti-reflective coating such as SiNx on the front side of the silicon wafer is required to improve the light-trapping effect.

Compared with conventional silicon solar cells, the surface recombination at the front surface has a more significant effect on the performance of IBC ones because of the greater distance between front surface and p-n junction at the rear side [1]. A better front surface passivation layer such as n+ front surface field that can repel the minority carries at the front surface is required to reduce the surface recombination loss.

The emitter contact fraction could also affect Jsc. The emitter contact fraction corresponds to the ratio between the PEDOT:PSS layer area and its electrode area. Due to resolution limitations, it was impossible to completely cover the PEDOT region of IBC cells with silver ink contact. There are simulated and experimental results that confirm that this parameter has a great impact on solar cell performances, including Jsc and recombination losses [34]. This issue can potentially be solved by applying other types of inkjet printing techniques, such as those used in the piezoelectric drop-on-demand inkjet printer, which can also potentially optimize the thickness of the PEDOT:PSS layer.

## 4. Summary

In this work, inkjet printing was demonstrated for the fabrication of interdigitated circuit solar cells. All key layers, including PEDOT:PSS films with co-solvents, TiO_2_ suspension and metal contacts, were printed on a textured silicon substrate, which means fully printed IBC structure hybrid solar cells were successfully fabricated with only one inkjet printer. Although the thickness of the PEDOT:PSS layer is in the order of a micrometer, with the addition of co-solvent and surface passivation the best short-circuit current is 19.2 mA/cm^2^, which indicates that there is a need to determine the processing window for the PEDOT:PSS film fabrication. The recombination loss at the PEDOT:PSS/silicon interface appears to be the primary issue existing for this IBC structure solar cell. Adding co-solvent and surface passivation significantly reduced the losses and enhanced the Jsc, and, as a result, improved the fill factor and efficiency of the devices. To further improve the performance of the devices, a proper anti-reflective coating and passivation layer on the front side is required. Also, an improved metal contact ink is needed to improve the contact resistivity between the PEDOT:PSS layer and the metal contact. Although PEDOT:PSS thickness has less influence on the hybrid IBC solar cells compared to the front-contact hybrid solar cells, due to the low vertical hole transport efficiency of PEDOT:PSS, a thinner PEDOT layer is still favorable. This issue can potentially be addressed by applying other types of inkjet printing techniques, such as those of the piezoelectric drop-on-demand inkjet printer.

## Figures and Tables

**Table 1:** Electrical performance of test structures (the layers and the Ag metal contacts are printed and textured Si wafers are used without BSF unless otherwise specified).

	Open-Circuit Voltage (V)	Short-Circuit Current (mA/cm^2^)	Fill Factor	Power Conversion Efficiency (%)
IBC, 0.3 mm interval PEDOT, Figure 2a,d	0.231	0.7	15.7	0.0
IBC, 0.3 mm interval PEDOT, Figure 2b,e	0.284	3.07	28.9	0.3
IBC, 0.35 mm interval PEDOT, Figure 2c,f	0.461	3.5	55.0	0.8
IBC, PEDOT with 7% DMSO and 0.5% FS-3100	0.557	13.0	31.3	2.3
Front-contact, with only evaporated metal contact	0.044	2.5	34.8	0.0
Front-contact, with spin-coated TiO_2_ and evaporated metal contact	0.431	2.5	32.9	0.3
IBC, with PEDOT (without co-solvent)	0.284	3.7	28.9	0.3
IBC, with PEDOT (without co-solvent) and TiO_2_	0.647	3.1	40.4	0.8
IBC, no passivation, PEDOT ink (with co-solvent)	0.557	13.0	31.3	2.3
IBC, BQ/ME passivation, PEDOT ink (with co-solvent)	0.559	19.1	31.9	3.4
IBC, no passivation, PEDOT ink (with co-solvent) and TiO_2_ ink	0.690	9.91	39.9	2.7
IBC, BQ/ME passivation, PEDOT ink (with co-solvent) and TiO_2_ ink	0.687	19.2	34.0	4.5
Front-contact, Ag/PEDOT:PSS/Si/BSF/Al (both metal contacts are evaporated, PEDOT is printed)	0.578	23.7	56.6	7.8
Front-contact, Ag/PEDOT:PSS/Si/BSF/Al (Al is evaporated, Ag contact and PEDOT are printed)	0.576	17.6	49.7	5.1

## Data Availability

The original contributions presented in this study are included in the article. Further inquiries can be directed to the corresponding author.

## Abbreviations

The following abbreviations are used in this manuscript:

PEDOT:PSS	Poly(3,4-ethylenedioxythiophene)-polystyrene sulfonate
IBC	Interdigitated back-contact
